# Symbiotic N_2_ Fixation and Grain Yield of Endangered Kersting's Groundnut Landraces in Response to Soil and Plant Associated *Bradyrhizobium* Inoculation to Promote Ecological Resource-Use Efficiency

**DOI:** 10.3389/fmicb.2018.02105

**Published:** 2018-09-11

**Authors:** Mustapha Mohammed, Sanjay K. Jaiswal, Elias N.K. Sowley, Benjamin D. K. Ahiabor, Felix D. Dakora

**Affiliations:** ^1^Department of Crop Sciences, Tshwane University of Technology, Pretoria, South Africa; ^2^Department of Chemistry, Tshwane University of Technology, Pretoria, South Africa; ^3^Department of Agronomy, University for Development Studies, Tamale, Ghana; ^4^CSIR-Savanna Agricultural Research Institute, Tamale, Ghana

**Keywords:** *B* value, δ^15^N, %Ndfa, N-fixed, SSR markers, neglected and under-utilized legume

## Abstract

Kersting's groundnut (*Macrotyloma geocarpum* Harms) is a neglected, endangered food and medicinal legume in Africa. Efforts to harness the benefits of the legume-rhizobia symbiosis have focused on few major legumes to the neglect of underutilized ones such as Kersting's groundnut. This study assessed plant growth, N-fixed and grain yield of five Kersting's groundnut landraces in response to inoculation with *Bradyrhizobium* strain CB756 at two locations in the Northern Region of Ghana. The transferability of cowpea-derived Simple Sequence Repeat (SSR) markers to Kersting's groundnut was also assessed. The symbiotic results revealed significant variation in nodulation, shoot biomass, δ^15^N, percent N derived from fixation, amount of N-fixed and soil N uptake. The cross-taxa SSR primers revealed monomorphic bands with sizes within the expected range in all the Kersting's groundnut landraces. The results of the aligned nucleotide sequences revealed marked genetic variability among the landraces. Kersting's groundnut was found to be a low N_2_-fixer, with 28–45% of its N derived from fixation at Nyankpala and 15–29% at Savelugu. Nitrogen contribution was 28–50 kg N-fixed·ha^−1^ at Nyankpala, and 12–32 kg N-fixed·ha^−1^ at Savelugu. Uninoculated plants of the Kersting's groundnut landraces Puffeun, Dowie, Sigiri and Boli, respectively, contributed 22, 16, 13, and 15 kg N-fixed·ha^−1^ from symbiosis at Savelugu as opposed to 89, 82, 69, and 89 kg N·ha^−1^ from soil. Landrace Puffeun was highly compatible with the introduced strain CB756 if based on δ^15^N and %Ndfa values, while Dowie, Funsi and Boli showed greater compatibility with native rhizobia in Ghanaian soils. The unimproved Kersting's groundnut in association with soil microsymbionts could produce grain yield of 1,137–1,556 kg ha^−1^ at Nyankpala, and 921–1,192 kg ha^−1^ at Savelugu. These findings suggest the need for further work to improve the efficiency of the Kersting's groundnut-rhizobia symbiosis for increased grain yield and resource-use efficiency in cropping systems.

## Introduction

The symbiosis between legumes and rhizobia often result in the formation of root nodules in which atmospheric N_2_ is reduced into NH_3_ for plant growth, improvement in soil fertility, and healthy ecosystem functioning (Nyemba and Dakora, 2010; Mohale et al., 2014). Legumes are therefore the best candidates for sustainable crop production, especially in nutrient-poor soils (Zahran, 1999; Siddique et al., 2012). Most grain legumes, including cowpea, soybean, groundnut and Bambara groundnut are known to meet a greater part of their N nutrition from atmospheric N_2_ fixation (Herridge and Peoples, 1990; Belane and Dakora, 2010; Mohale et al., 2014; Mokgehle et al., 2014; Mapope and Dakora, 2016). Although Kersting's groundnut (*Macrotyloma geocarpum*, Harms.) is reported to nodulate with rhizobia in sand culture under glasshouse conditions (Dakora et al., 1992), its N_2_-fixing potential under field conditions has not been assessed so far. Inoculating legumes with superior inoculant strains can result in increased nodulation, N_2_ fixation and grain yield. However, the presence of large populations of native/resident soil rhizobia can outcompete the introduced strain and cause inoculation failure. Thus, the success of rhizobial inoculation in the field is often measured by the extent of nodulation, N_2_ fixation, plant growth and grain yield when compared to uninoculated control (Kyei-Boahen et al., 2017). Of the many techniques available, the ^15^N natural abundance has been very successful in quantifying N_2_ fixation by grain legumes under field conditions (Mohale et al., 2014; Mapope and Dakora, 2016; Gyogluu et al., 2017).

The Kersting's groundnut is geocarpic and grown in the semi-arid areas of West Africa, usually by older farmers. In Northern Ghana, it is grown for superstitious uses and its cultivation is surrounded by myths (Amuti, 1980). For example, among the Dagomba tribe of Northern Ghana, it is believed that opening the leaves of Kersting's groundnut plants to sight flowering or podding can result in one's secretes being revealed. In Central Benin, Kersting's groundnut has a significant economic value for most households (Assogba et al., 2016). The seeds contain up to 21.3% protein (Ajayi and Oyetayo, 2009; Dansi et al., 2012), with high levels of essential amino acids such as lysine, phenylalanine and methionine (Aremu et al., 2006). Despite these attributes, low grain yield, taste preferences and neglect by research have contributed to its reduced cultivation even though yields of up to 1,876 and 3,840 kg·ha^−1^ have been respectively recorded in Ghana and Benin (Bayorbor et al., 2010; Assogba et al., 2016).

Currently, Kersting's groundnut landraces locally grown by farmers exhibit different seed coat colors. However, there is so far no report on the genetic characterization of these landraces. Molecular characterization is an important tool for studying genetic diversity in the breeding and conservation of plant species (Jaiswal et al., 2013), and can also be used in the genetic improvement of legumes to strengthen biological N_2_ fixation technologies. Microsatellites or Simple sequence repeats (SSRs) have become potent markers for diversity studies due to their abundance, high degree of polymorphism and co-dominant properties (Tautz and Renz, 1984). The development of species-specific SSR primers is expensive and time-consuming, thus causing its limited use. However, the homology of flanking regions of SSRs often allows cross species/genus amplification (transferability) in the same taxa (Soldati et al., 2014). Therefore, the transferability of SSR markers is a potent tool for breeding and conservation studies. An earlier study reported effective nodulation of Kersting's groundnut by *Bradyrhizobium* strain CB756, a strain known to effectively nodulate cowpea (Dakora et al., 1992). In addition, other studies have reported on cowpea synteny to other legumes such as soybean and clover (Lucas et al., 2011, 2013), which motivated the use of cowpea SSR primers to characterize Kersting's groundnut in this study.

Furthermore, the amount of N-fixed by this legume under field conditions is not known. Moreover, the response of Kersting's groundnut to rhizobial inoculation in the field also remains unknown, and the possible presence of compatible and efficient resident or native rhizobia in soils has not been explored. In a world that currently relies on few staple crops for food and nutritional security (Mayes et al., 2012), it is important to conserve and improve the potential yield of under-utilized legumes such as Kersting's groundnut which could serve as suitable crops in sustainable farming systems. The aim of this study was to assess N_2_ fixation and grain yield in field-grown Kersting's groundnut landraces nodulated by resident soil microsymbionts and *Bradyrhizobium* strain CB756 inoculant, using the ^15^N natural abundance technique. The cross transferability of cowpea SSR markers to the crop was also explored in this study.

## Materials and methods

### Description of experimental sites

**Figure 1:**
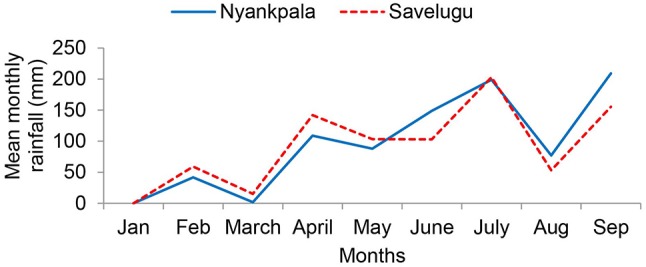
Rainfall distribution at Nyankpala and Savelugu in the Northern Region, Ghana, during the 2012 cropping season.

The study was conducted at Nyankpala (latitude 9.404, longitude −0.982, and altitude of 162 m) and Savelugu (latitude 9.569, longitude −0.830, and altitude of 162 m) in the Northern Region of Ghana. The sites fall within the Guinea Savanna agro-ecological zone. Rainfall in the region is unimodal (800–1,200 mm), and occurs between April and October each year (Owusu and Waylen, 2013). The rainfall distribution during the experimental period is illustrated in Figure 1. The Nyankpala site was fallowed for a long time with no history of fertilization, while the Savelugu site was planted to maize and fertilized with NPK in the previous year. The two sites had no history of *Rhizobium* inoculation. Before planting, soil samples were randomly collected from 20 different areas (0 – 20 cm) within the experimental plots of each field, pooled, and subsamples analyzed for chemical properties such as pH (H_2_O), texture, organic C (Walkley and Black, 1934), total N (using Dry combustion method), P using Bray-2 (Bray and Kurtz, 1945), and K, Na, Ca, Mg, S, and cation exchange capacity (CEC; using ammonium acetate method) according to Rowell (1994) (Table 1).

**Table 1:** Physico-chemical properties of soils collected from the two study locations in 2012.

	**pH (H_2_O)**	**SOC**	**N**	**Sand**	**Silt**	**Clay**	**P**	**Na**	**K**	**Ca**	**Mg**	**CEC**
**Locations**		**%**					**mg**·**kg**^**−1**^					**Cmol·kg^−1^**
Nyankpala	5.6	0.36	0.04	68	20	12	2.0	17.7	40.6	309	81.2	2.8
Savelugu	5.4	0.55	0.06	68	20	12	2.1	21.4	48.8	338	103.0	4.2

### Origin of kersting's bean seeds used in this study

**Figure 2:**
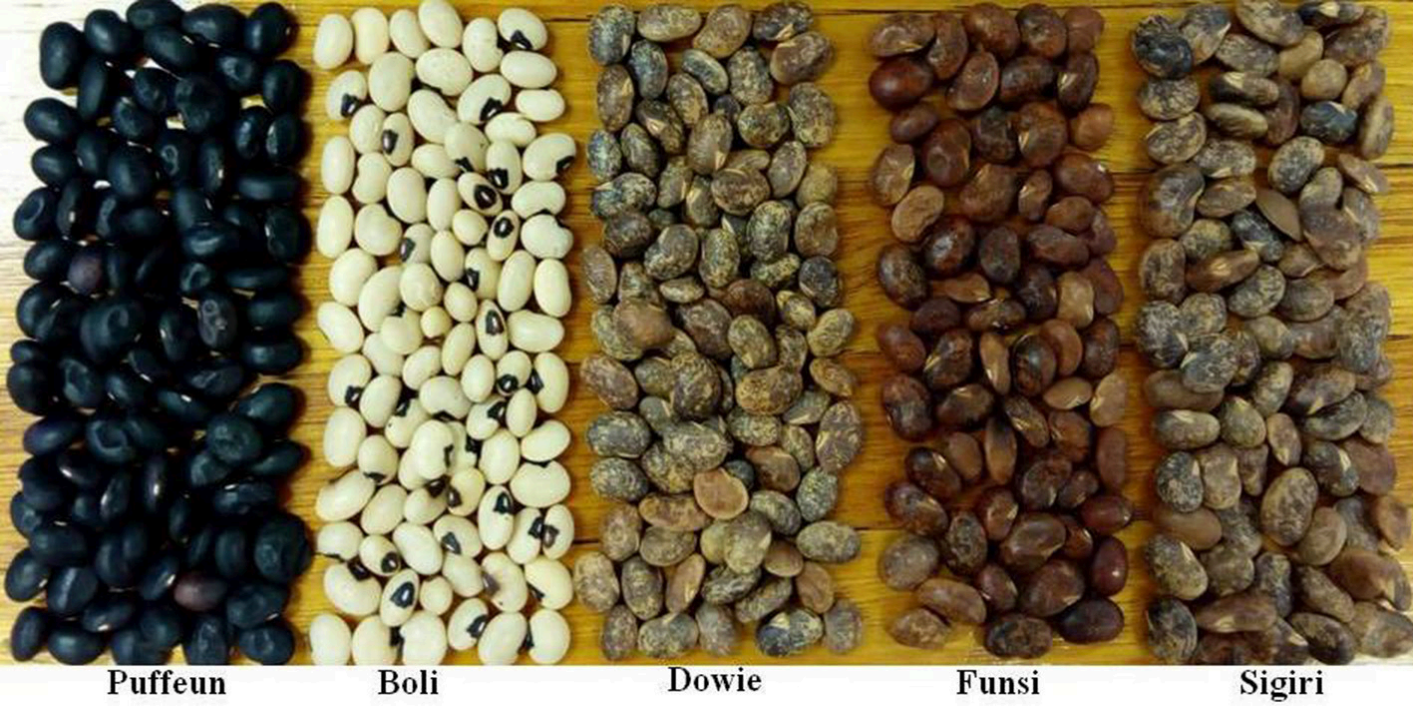
Kersting's groundnut seeds showing different seed coat colors.

Five Kersting's groundnut landraces [Puffeun (Black), Boli (White), Dowie (mottled), Funsi (Red), and Sigiri (Mottled)] (Figure 2) were obtained from the Agronomy Department of the University for Development Studies at Nyankpala, Ghana. These landraces were earlier collected from different villages in Northern Ghana (Bayorbor et al., 2010).

### DNA extraction, SSR primers and PCR amplifications

Seeds of each Kersting's groundnut landrace were planted in separate plastic pots (diameter = 15 cm) containing sand. After 3 weeks of growth in a naturally lit glasshouse, the newly emerging leaves of each landrace were collected for DNA extraction using Invisorb Spint Plant mini kit (Stratec, Germany) according to the manufacturer's procedure. The transferability of 12 cowpea-derived SSR markers (CLM0014, CLM0018, CLM0022, CLM0028, CLM0030, CLM0033, CLM0034, CLM0037, CLM0040, CLM0044, VuUGM80, and VuUGM87; Gupta and Gopalakrishna, 2010; Xu et al., 2010) to Kersting's groundnut was tested. DNA amplifications were carried out in 25 μL reaction mixtures, each containing a final concentration of 40–50 ng template DNA, 3 μl (5 ×) PCR buffer (Bioline), 1 μl (10 pM) of each primer and 0.1 μl (5U) taq polymerase (Bioline USA) using different annealing temperatures (Table S1) for each primer pairs under the following PCR cycling conditions: 95 ^o^C for 3 min, 36 × (denaturation at 94°C for 30 s. annealing at 50.1–56.1°C for 30 s, extension at 72°C for 30 s) and final extension at 72°C for 5 min. The amplified products were separated on 2.5% agarose gel electrophoresis stained with ethidium bromide. The sizes of bands were determined by comparison to a 100 bp DNA ladder (Bioline, USA).

### Sequencing of SSR amplified products

The SSR amplified products were purified (Rapid PCR Cleanup Enzyme Set, New England Biolabs, USA) and sequenced (Macrogen, Netherland). The quality of all sequences was checked using BioEdit 7.0.0 software (Hall, 2004). Sequences were then aligned using MEGA 6.0 software (Tamura et al., 2013).

### Field experimental design and planting

The experimental treatments included the five Kersting's groundnut landraces and two levels of inoculation (uninoculated and inoculated with *Bradyrhizobium* strain CB756). The trial was laid out in a randomized complete block design with four replicates for each treatment combination per site. Each plot measured 2 × 3 m. Two seeds were planted per hole (20 cm between plants and 60 cm between rows) on 24 and 25th June 2012 at Nyankpala and Savelugu, respectively.

### Seed inoculation

Seeds were inoculated with a peat-based inoculant of *Bradyrhizobium* strain CB756 (10^8^ rhizobial cells·gpeat^−1^) at a rate of 10 g inoculant per kg seed. Inoculated seeds were allowed to dry under shade for few minutes before planting. The uninoculated control treatments were planted first, followed by the inoculated treatments to avoid contamination. The plots were kept weed-free by hand hoeing when necessary.

### Plant sampling and processing

Five plants were randomly sampled at flowering, which coincided with 52 and 53 days after planting (DAP) at Nyankpala and Savelugu, respectively, to assess nodulation and measure N_2_ fixation. The plants were carefully dug up and separated into shoots and nodulated roots. The nodules were plucked from the roots. The shoots and nodules were separately oven-dried at 65°C for 48 h, weighed and ground (0.85 mm sieve size). Non-legume reference plants which were sampled from plots alongside the landraces were similarly oven-dried (65°C for 48 h), weighed and ground (0.85 mm) for ^15^N isotopic analysis. The mean δ^15^N of the reference plants for each site replaced the δ^15^N of soil N taken up by the test legume at the site (Unkovich et al., 2008).

### ^15^N/^14^N isotopic analysis

About 2.0–2.5 mg of ground legume and reference plant samples were fed into a Carlo Erba NA1500 elemental analyzer (Fisons Instruments SpA, Strada, Rivoltana, Italy) coupled to a Finnigan MAT252 mass spectrometer (Fisons Instrument SpA, Strada, Rivoltana, Italy) via conflo II open-split device to measure their respective ^15^N/^14^N isotopic composition (Mapope and Dakora, 2016). The δ^15^N of the shoot samples was computed as Unkovich et al. (2008):

(1) δ15N (‰)=[N15/N14]sample−[N15/N14]atm[N15/N14]atm×1,000

Where ^15^N/^14^N_sample_ is the abundance ratio of ^15^N and ^14^N in the sample and ^15^N/^14^N_atm_ is the abundance ratio of ^15^N and ^14^N in the atmosphere. The %N and %C of shoot samples were obtained directly from the mass spectrometer, and shoot C/N ratio computed as the ratio of C to N.

### Shoot N content

The N content of shoot samples was calculated as the product of shoot dry matter (DM) and %N (Pausch et al., 1996).

### Determination of B value

The B value was determined in glasshouse studies by inoculating two Kersting's groundnut landraces (Puffeun and Funsi) with a commercial inoculant of *Bradyrhizobium* strain CB756. This strain was previously used in studies of N_2_ fixation in Kersting's groundnut (Dakora et al., 1992). Before planting, seeds of the two landraces were surface-sterilized by immersion in 95% ethanol for 5 s, and then in 3.5% sodium hypochlorite for 3 min, followed by rinsing five times with sterile distilled water (Somasegaran and Hoben, 1994). Two seeds were sown aseptically in sterile sand contained in plastic pots (diameter ≈ 20 cm). Three replicate pots were used per landrace. After germination, the seedlings were inoculated with 1 ml yeast mannitol broth suspension of *Bradyrhizobium* strain CB756 (≈1 × 10^7^-1 × 10^8^ cells/ml). The plants were supplied with N-free nutrient solution (Broughton and Dilworth, 1971), and when necessary, with sterile distilled water. The plants were harvested at 52 DAP, separated into shoots and nodulated roots, and oven-dried at 65°C for 48 h. The shoot and root samples were then separately ground (0.85 mm sieve) for each landrace and used for ^15^N isotopic analysis. Whole-plant δ^15^N values (shoots + roots) were calculated as the average δ^15^N values of plant organs (shoots and roots), weighted to their respective N contents (Robinson, 2001; Nebiyu et al., 2014).

(2) $$\begin{array}{r} \delta^{15}{\text{N}}_{\text{wholeplant}}=\frac{\delta^{15}{\text{N}}_{\text{shoot}}\times \text{Nconten}{\text{t}}_{\text{shoot}}+\delta^{15}{\text{N}}_{\text{root}}\times \text{Nconten}{\text{t}}_{\text{root}}}{\text{Nconten}{\text{t}}_{\text{shoot}}+\text{Nconten}{\text{t}}_{\text{root}}} \end{array}$$

The average δ^15^N value of shoots of the two landraces (Puffeun and Funsi) was used as the B value in estimating %Ndfa of the test landraces.

### Percent N derived from fixation (%Ndfa)

The %Ndfa of the test legumes was calculated as (Shearer and Kohl, 1986):

(3) $$\begin{array}{r} \%\text{Ndfa}=\frac{\delta^{15}{\text{N}}_{\text{ref}}-\delta^{15}{\text{N}}_{\text{leg}}}{\delta^{15}{\text{N}}_{\text{ref}}-\text{B}}\times 100 \end{array}$$

Where δ^15^N_ref_ is the ^15^N natural abundance of reference plant, δ^15^N_leg_ is the ^15^N natural abundance of legume plant, and the B value is the ^15^N natural abundance of the shoot of Kersting's groundnut plant completely dependent on atmospheric N_2_ for its N nutrition. The mean δ^15^N of all reference plants sampled from each study site was used to estimate the %Ndfa of landraces.

### Amount of N-fixed and soil N uptake

The amount of N-fixed in Kersting's groundnut plants was calculated as (Maskey et al., 2001):

(4) $$\begin{array}{r} \text{N}-\text{fixed}=\left(\frac{\text{\%Ndfa}}{100}\right)\times \text{N content of Kersting}\text{'}s\;groundnut\;shoots \end{array}$$

The soil N uptake by Kersting's groundnut was calculated as the difference between total shoot N content and the amount of N-fixed.

### Grain yield

At physiological maturity, 10 plants were harvested from the three inner rows of each plot to determine grain yield. Pods were first separated from plants, air-dried to 15% moisture content, and threshed to obtain the grain from pods. The dried seeds were weighed and the grain yield determined per hectare using plant density.

### Statistical analysis

The data collected from the field studies were tested for normal distribution before being subjected to analysis of variance using STATISTICA version 10 (Statsoft Inc, 2011). A two-way analysis of variance (ANOVA) was used to compare means of inoculation × landrace interaction at each site, while a three-way ANOVA was used to compare treatment means across sites for inoculation and landraces. Means that showed significant differences were separated using Duncan's multiple range test at *p* ≤ 0.05. Correlation analyses were performed to determine the relationships between measured parameters.

## Results

### Soil chemical properties and rainfall distribution of the study sites

The soils from Nyankpala and Savelugu were both sandy loam in texture. Although soils from both sites were generally low in nutrients, the levels were slightly higher in soils at Savelugu when compared to Nyankpala (Table 1). Total rainfall from the beginning of the cropping season to plant harvest was 828 mm and 880 mm at Nyankpala and Savelugu, respectively. However, the total rainfall from sowing to plant sampling was 638 mm at Nyankpala and 514 mm at Savelugu (Figure 1).

### Cross-genus transferability of cowpea derived SSR markers to kersting's groundnut

**Figure 3:**
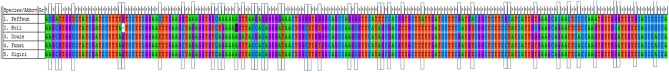
Aligned nucleotide sequences of genomic regions of Kersting's groundnut landraces generated by CLM0044 primer.

Out of the 12 cross-taxa SSR primers used in this study, eight (66%) showed monomorphic bands with sizes within the expected range in all the Kersting's groundnut landraces (Table S1). Primer VuUGM87 yielded non-specific bands with the landraces tested. However, the SSR primers CLM0018, CLM0028 and VuUGM80 did not show any amplification. To assess the genetic variability among landraces at the nucleotide sequence level, amplified products of primer CLM0044 were sequenced. The SSR sequence analysis revealed different “TT,” “GG,” “CT,” and “AA” motif types with 15, 7, 10, and 9 times of repeats, respectively. The aligned sequence results revealed the presence of genetic variation among the test landraces, with nucleotide differences occurring at different positions (Figure 3). The landrace Puffeun had 36 nucleotide mismatch differences, and was the most genetically distinct among the test landraces (Figure 3).

### The δ^15^N values of non-N_2_-fixing reference plants

The δ^15^N of non-legume reference plants ranged from +2.93%0 to +5.08%0 for Nyankpala and +1.37%0 to +4.18%0 for Savelugu (Table 2). The combined mean δ^15^N values used to estimate %Ndfa were +3.95%0 for Nyankpala and +3.04%0 for Savelugu (Table 2).

**Table 2:** The δ^15^N of reference plants sampled from Nyankpala and Savelugu in the Northern region of Ghana in 2012, for the estimation %Ndfa in Kersting's groundnut.

**Locations**	**Common names**	**Botanical names**	**δ^15^N (%0)**
**NYANKPALA**			
	Goat weed	*Ageratum conyzoides*	3.85
	Garaji	*Brachiaria lata*	5.08
	Finger grass	*Chloris pilosa*	4.12
	Jew's mallow	*Corchorus olitorius*	3.98
	Bermuda grass	*Cynodon dactylon*	2.93
	Mint weed	*Hyptis suaveolens*	3.50
	Water primrose	*Ludwigia abyssinica*	3.66
	Willow primrose	*Ludwigia decurrens*	3.77
	Rice grass	*Paspalum scrobiculatum*	4.66
	Mean		+3.95 ± 0.63
**SAVELUGU**			
	Goat weed	*Ageratum conyzoides*	3.53
	Garaji	*Brachiaria lata*	4.18
	Finger grass	*Chloris pilosa*	3.54
	Jew's mallow	*Corchorus olitorius*	1.37
	Bermuda grass	*Cynodon dactylon*	2.48
	Mint weed	*Hyptis suaveolens*	3.43
	Water primrose	*Ludwigia abyssinica*	3.42
	Willow primrose	*Ludwigia decurrens*	3.34
	Rice grass	*Paspalum scrobiculatum*	2.09
	Mean		+3.04 ± 0.88

*The mean δ^15^N (values ± SD) of reference plants at each location was used to calculate %Ndfa by Kersting's groundnut plants in that location*.

### The δ^15^N values of kersting's beans fully dependent on symbiotic N_2_ fixation (B value)

Two Kersting's bean landraces (namely, Puffeun and Funsi) were used to determine the B value. As shown in Table 3, the B value of shoots and roots of the 52-d-old Kersting's groundnut plants was similar for both landraces. However, the B value of whole plants was significantly higher in Funsi than Puffeun (Table 3). In this study, the average δ^15^N value of shoots was used to estimate %Ndfa.

**Table 3:** B values (or organ δ^15^N) of two Kersting's groundnut landraces which were solely dependent on atmospheric N_2_ fixation for their N nutrition.

**Landrace**	**Root δ^15^N**	**Shoot δ^15^N**	**Whole plant δ^15^N**
	%0	%0	%0
Puffeun	−2.60 ± 0.30*a*	−3.68 ± 0.49*a*	−2.65 ± 0.12*b*
Funsi	−3.31 ± 0.08*a*	−3.99 ± 0.01*a*	−1.91 ± 0.01*a*
*F-statistics*	5.24^ns^	0.40^ns^	39.36**

*Plants were sampled at 52 DAP*.

Values (Mean ± S.E.) with dissimilar letters in a column are significantly different at p = 0.01 (**); ns, not significant.

### Plant growth, symbiotic N nutrition and grain yield of kersting's bean landraces

A 2-Way ANOVA analysis of data for parameters measured at each experimental site revealed differences in main effects, as well as in landrace × inoculation interaction effects (Table 4). At both sites, the main effect of landrace was significant for shoot biomass, nodule dry matter, N content, and all symbiotic parameters (δ^15^N, %Ndfa, amount of N-fixed) as well as soil N uptake. Although grain yield varied among the landraces grown at Nyankpala, those at Savelugu showed similar grain yields (Table 4). On the other hand, the main effect of inoculation was significant for shoot DM, nodule DM, N content and N-fixed at both test locations (Table 4).

**Table 4:** A two-way ANOVA of plant growth, nodulation, symbiotic parameters and grain yield of five Kersting's groundnut landraces inoculated with or without *Bradyrhizobium* strain CB756 at Nyankpala and Savelugu in Northern Ghana in 2012.

**Location/treatment**	**Shoot DM (g plant^−1^)**	**Nodule DM (mg plant^−1^)**	**N conc'n (%)**	**N content (mg plant^−1^)**	**C/N (g g^−1^)**	**δ^15^N (%0)**	**Ndfa (%)**	**N fixed (kg ha^−1^)**	**Soil N uptake (kg ha^−1^)**	**Grain yield (kg ha^−1^)**
**NYANKPALA**										
**Landrace**
Puffeun	20.40 ± 0.99b	5.96 ± 0.38c	2.77 ± 0.11a	567.09 ± 37.68c	14.27 ± 0.44a	1.03 ± 0.23c	37.46 ± 2.96a	35.51 ± 3.74b	59.00 ± 4.73c	*1, 484.00*±82.60ab
Dowie	22.58 ± 0.91b	7.01 ± 1.10bc	2.92 ± 0.14a	653.42 ± 26.25bc	14.79 ± 0.49a	±0.19bc	35.15 ± 2.41ab	38.16 ± 2.76b	70.75 ± 4.09bc	*1, 136.65*±65.73c
Funsi	27.04 ± 2.38a	7.36 ± 1.17b	2.86 ± 0.10a	773.93 ± 72.78a	14.29 ± 0.62a	1.07 ± 0.15c	37.02 ± 1.99a	46.30 ± 2.50a	82.68 ± 10.18ab	*1, 265.94*±84.99bc
Sigiri	26.36 ± 0.53a	5.97 ± 0.54c	2.94 ± 0.10a	777.20 ± 35.47a	14.47 ± 0.43a	1.65 ± 0.12a	29.57 ± 1.51c	38.18 ± 2.48b	91.35 ± 4.92a	*1, 232.67*±100.35c
Boli	22.76 ± 0.77b	8.92 ± 1.55a	3.04 ± 0.15a	689.16 ± 30.70ab	14.71 ± 0.70a	±0.13b	32.90 ± 1.72b	37.57 ± 2.09b	77.29 ± 4.67ab	*1, 556.21*±81.72a
**Inoculation**
Uninoculated	22.12 ± 0.65b	7.94 ± 0.77a	2.94 ± 0.07a	653.06 ± 27.75b	14.47 ± 0.32a	1.30 ± 0.12a	34.00 ± 1.53a	36.56 ± 1.83b	72.28 ± 4.07a	*1, 316.57*±68.86a
Inoculated	25.53 ± 1.08a	6.14 ± 0.49b	2.87 ± 0.07a	731.26 ± 33.74a	14.55 ± 0.35a	1.24 ± 0.11a	34.84 ± 1.42a	41.73 ± 1.75a	80.15 ± 4.72a	*1, 353.62*±55.17a
**F statistics**
*Landrace (L)*	12.38^***^	7.47^***^	*0.72ns*	5.61^**^	*0.18ns*	8.90^***^	8.90^***^	4.61^**^	6.18^***^	5.13^**^
*Inoculation (I)*	23.10^***^	20.37^***^	*0.42ns*	5.52^*^	*0.03ns*	*0.74ns*	*0.74ns*	8.93^**^	*3.20ns*	*0.28ns*
*L × I*	9.94^***^	35.12^***^	*1.29ns*	3.25^*^	*0.79ns*	27.24^***^	27.24^***^	8.27^***^	5.36^***^	*2.47ns*
**SAVELUGU**										
**Landrace**
Puffeun	16.52 ± 1.71b	4.85 ± 0.25d	2.89 ± 0.26a	504.02 ± 93.19b	11.69 ± 0.66a	1.59 ± 0.05ab	21.04 ± 0.70ab	17.10 ± 2.80b	66.90 ± 12.74b	*1, 059.2*±143.2a
Dowie	16.22 ± 1.32b	6.28 ± 1.45c	3.03 ± 0.27a	491.81 ± 59.64b	11.83 ± 0.80a	1.64 ± 0.14ab	20.36 ± 1.98ab	15.86 ± 1.57b	66.11 ± 9.02b	931.5 ± 177.2a
Funsi	23.13 ± 1.53a	7.68 ± 0.34b	3.22 ± 0.19a	731.39 ± 38.06a	11.37 ± 0.67a	1.45 ± 0.15b	23.15 ± 2.22a	27.63 ± 2.23a	94.27 ± 6.87a	921.5 ± 154.8a
Sigiri	14.83 ± 0.59b	10.77 ± 1.16a	2.96 ± 0.28a	432.77 ± 37.50b	11.56 ± 0.59a	1.81 ± 0.11a	17.82 ± 1.58b	12.80 ± 1.57b	59.33 ± 5.40b	*1, 040.0*±183.5a
Boli	17.01 ± 1.81b	2.85 ± 0.31e	3.14 ± 0.21a	516.40 ± 40.79b	11.53 ± 0.47a	1.82 ± 0.11a	17.79 ± 1.54b	15.16 ± 1.59b	70.91 ± 6.07b	*1, 192.1*±139.9a
**Inoculation**
Uninoculated	18.91 ± 0.87a	4.95 ± 0.54b	3.18 ± 0.14a	604.01 ± 37.54a	11.72 ± 0.41a	1.71 ± 0.09a	19.30 ± 1.32a	19.77 ± 2.04a	80.91 ± 4.89a	*1, 105.1*±109.0a
Inoculated	16.17 ± 1.22b	8.03 ± 0.87a	2.91 ± 0.15a	466.54 ± 40.76b	11.47 ± 0.38a	1.61 ± 0.06a	20.77 ± 0.86a	15.65 ± 1.09b	62.11 ± 5.87b	952.6 ± 86.91a
**F statistics**
*Landrace (L)*	8.21^***^	73.34^***^	*0.35ns*	5.54^**^	*0.06ns*	3.77^*^	3.77^*^	10.86^***^	3.97^*^	*0.52ns*
*Inoculation (I)*	7.38^*^	97.25^***^	*1.76ns*	10.03^**^	*0.17ns*	*1.97ns*	*1.97ns*	6.94^*^	9.78^**^	*1.23ns*
*L × I*	5.30^**^	21.58^***^	*1.78ns*	*2.44ns*	*0.10ns0*	10.04^***^	10.04^***^	*2.38ns*	3.89^*^	*1.76ns*

Values (mean ± SE) with dissimilar letters in a column are significantly different at

* *p ≤ 0.05*,

** p ≤ 0.01, or

*** *p ≤ 0.001; ns, not significant*.

At Nyankpala, the landraces Funsi and Sigiri recorded much higher shoot DM, while nodule mass was higher in the landrace Boli, followed by Funsi and Dowie. However, together with Boli, the landraces with greater shoot DM also elicited greater shoot N content. Landraces Puffeun, Funsi and Dowie recorded lower δ^15^N values at Nyankpala, which resulted in greater %Ndfa by those landraces at the site. However, N-fixed rose in only Funsi due to its greater shoot DM (Table 4). Soil N uptake by the landraces closely mirrored their shoot N accumulation, with higher values recorded in Sigiri, Funsi and Boli. At Nyankpala, the landraces Boli and Puffeun produced the highest grain yield (1,556 and 1484 kg ha^−1^, respectively), while Dowie produced the lowest (1,137 kg ha^−1^; Table 4).

At Savelugu, landrace Funsi again recorded the highest shoot DM, while nodule DM was greater in Sigiri, followed by Funsi, and lowest in Boli (Table 4). With higher shoot DM, landrace Funsi also showed greater shoot N accumulation, while the others elicited lower but similar shoot N. Landrace Funsi showed the lowest shoot δ^15^N value at Savelugu, while Sigiri and Boli recorded the highest (Table 4). As a result, the landrace Funsi derived relatively more N from fixation, while Sigiri and Boli obtained the least N from symbiosis (Table 4). As found in Nyankpala, the amount of N-fixed as well as soil N uptake were also greater in the Funsi landrace than the other landraces at Savelugu (Table 4). The grain yield of all landraces was similar at Savelugu, with values ranging from 922 kg ha^−1^ for landrace Funsi to 1,192 kg ha^−1^ for Boli.

### Effect of landrace × inoculation interaction on plant growth and symbiotic N nutrition

**Figure 4:**
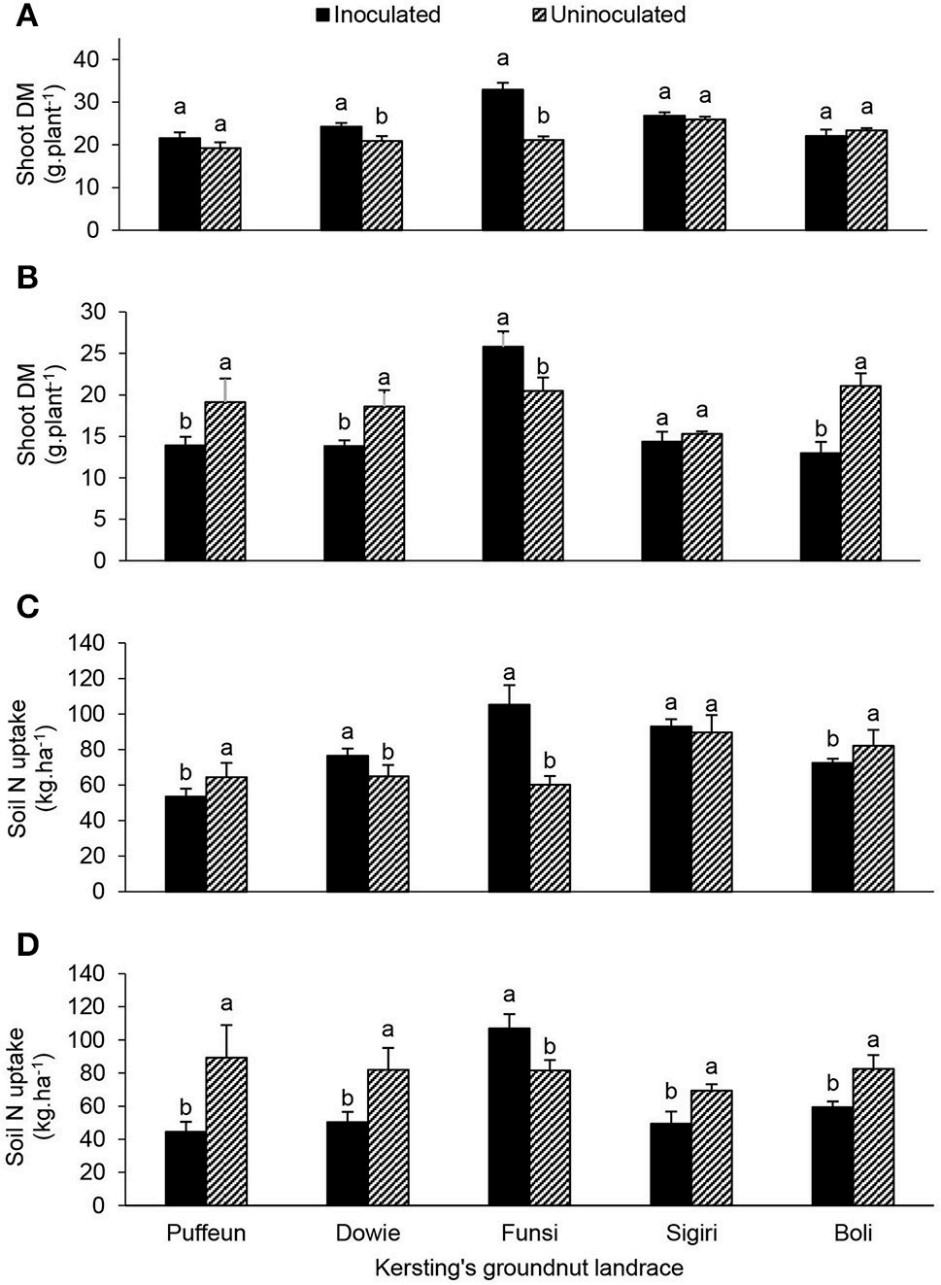
The interactive effect of landrace × inoculation on **(A)** shoot dry matter at Nyankpala, **(B)** shoot dry matter at Savelugu, **(C)** soil N uptake at Nyankpala, and **(D)** soil N uptake at Savelugu. For each landrace, bars with dissimilar letters are significantly different at *p* ≤ 0.05. Error bars represent S.E.

**Figure 5:**
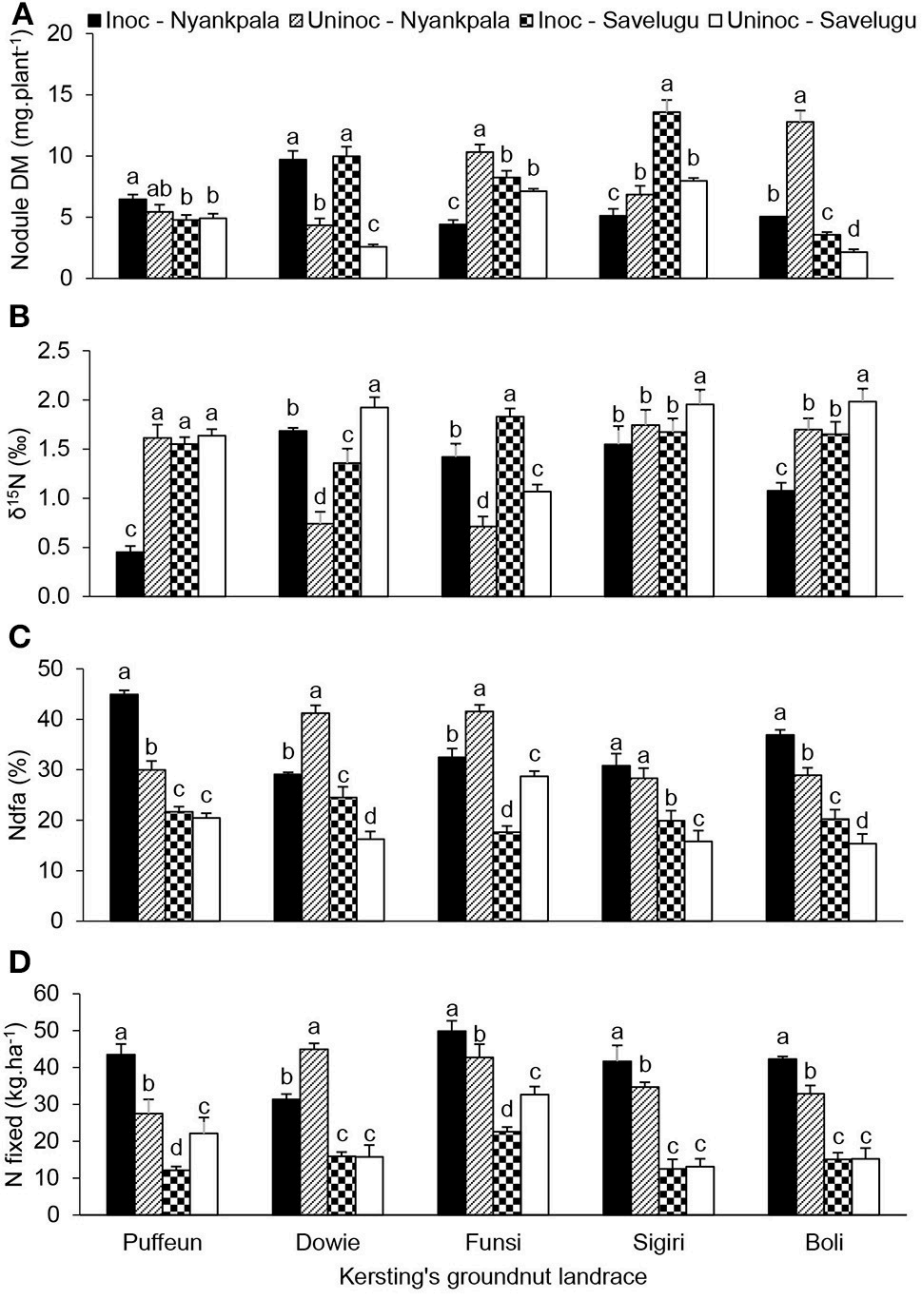
The interactive effect of landrace × inoculation × location on **(A)** nodule dry matter, **(B)** δ^15^N, **(C)** % Ndfa, and **(D)** N-fixed in field-grown Kersting's groundnut landraces. For each landrace, bars with dissimilar letters are significantly different at *p* ≤ 0.05. Error bars represent S.E.

At Nyankpala, the interactive effect of landrace × inoculation was significant for shoot DM, nodule DM, N content, δ^15^N, %Ndfa, N-fixed and soil N uptake (Table 4; Figures 4, 5). As shown in Figure 4A, bradyrhizobial inoculation markedly increased shoot DM in landraces Dowie and Funsi, but not in the other three landraces. However, although bacterial inoculation at Nyankpala also increased nodule DM in Dowie, the inoculated plants of the landraces Funsi, Sigiri and Boli had much lower values at that site (Figure 5A). However, inoculating the five landraces at Nyankpala increased the δ^15^N of Dowie and Funsi, but decreased those of Puffeun and Boli. But the δ^15^N of the landrace Sigiri was unaltered by inoculation at the site (Figure 5B). As a result, the percent N derived from fixation by inoculated plants was significantly higher in Puffeun and Boli, markedly lower in Dowie and Funsi, and unaltered in Sigiri relative to the uninoculated control (Figure 5C). The amount of N-fixed was however greater in shoots of all inoculated landraces except for Dowie, which recorded more N-fixed in shoots of the uninoculated control (Figure 5D). Soil N uptake was also higher in the inoculated plants of Dowie and Funsi, lower in Puffeun and Boli, and unaltered by inoculation in Sigiri (Figure 4C).

The landrace × inoculation interaction was also significant for shoot DM, nodule DM, δ^15^N, %Ndfa and soil N uptake at Savelugu (Table 4). Here, bradyrhizobial inoculation significantly decreased shoot DM in landraces Puffeun, Dowie and Boli, but increased it in Funsi (Figure 4B), even though nodule DM was greater in inoculated plants of Dowie and Boli (Figure 5A). With inoculation however, shoot δ^15^N values were markedly lowered in Dowie, Sigiri and Boli, increased in Funsi, but unaltered in Puffeun (Figure 5B). As a result, the percent N derived from fixation was much higher in inoculated plants of landraces Dowie, Sigiri and Boli, and lower in Funsi, but unaltered in Puffeun when compared to the uninoculated control (Figure 5C). As to be expected soil N uptake was lower in the inoculated Dowie, Sigiri and Boli landraces (Figure 4D), which derived greater N from symbiotic fixation (Figure 5C).

### Comparative analysis of plant growth, N nutrition and grain yield between study sites

A three-Way ANOVA revealed a significant effect of location on shoot DM, nodule DM, N content and all symbiotic parameters (δ^15^N, %Ndfa and N-fixed) as well as grain yield (Table S2). Overall, the shoot DM, nodule DM, shoot N content and C/N ratio of the landraces were much greater at Nyankpala (Table S2). However, with lower shoot δ^15^N and higher shoot DM, the plants at Nyankpala elicited greater %Ndfa and N-fixed when compared to those grown at Savelugu (Table S2). Overall, the grain yield of landraces was higher at Nyankpala (1,335 kg·ha^−1^) when compared to Savelugu (1,029 kg·ha^−1^; Table S2).

**Figure 6:**
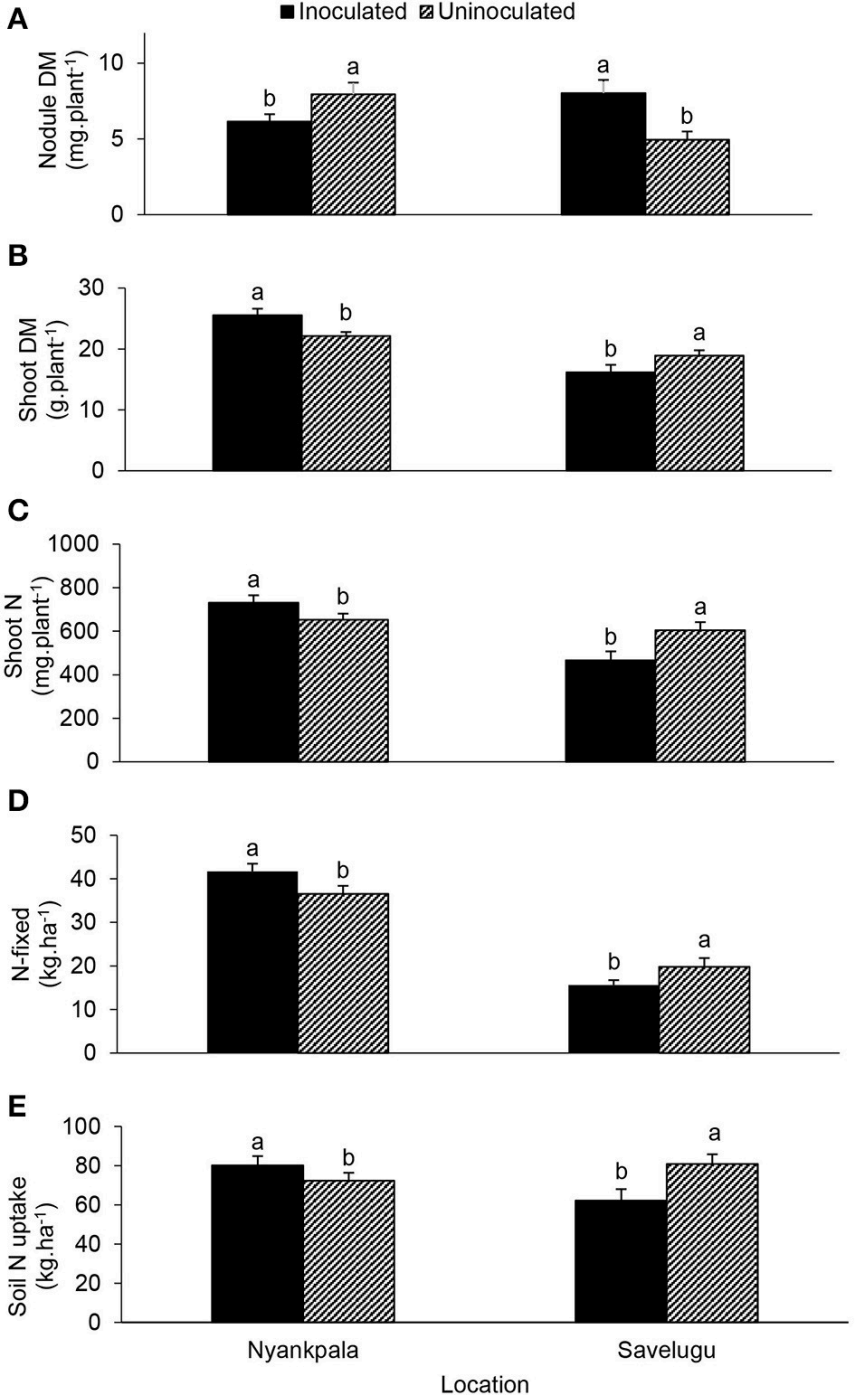
The interactive effect of inoculation × location on **(A)** nodule dry matter, **(B)** shoot dry matter, **(C)** shoot N content, **(D)** amount of N-fixed, and **(E)** soil N uptake of field-grown Kersting's groundnut planted in 2012. For each location, bars with dissimilar letters are significantly different at *p* ≤ 0.05. Error bars represent S.E.

From the interactive effect of inoculation × location (Figure 6), *Bradyrhizobium* application to Kersting's groundnut landraces increased shoot DM, shoot N content, N-fixed and soil N uptake at Nyankpala, even though nodule DM was reduced (Figures 6A–E). Conversely, despite an increase in nodule DM from inoculant application at Savelugu, shoot DM, N content, amount of N-fixed, and soil N uptake were much lower (Figures 6A–E).

**Figure 7:**
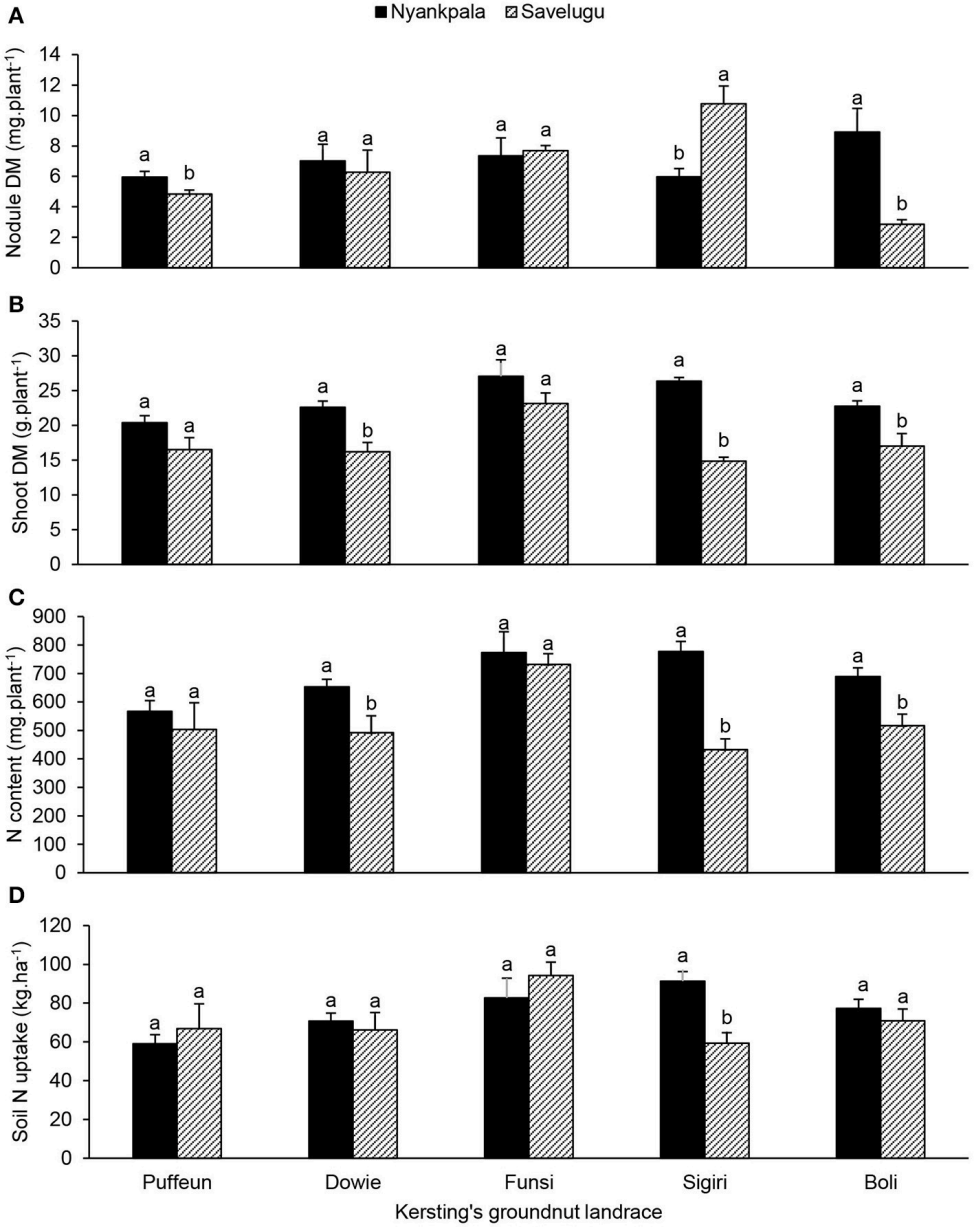
The interactive effect of landrace × location on **(A)** nodule dry matter, **(B)** shoot dry matter, **(C)** shoot N content, and **(D)** soil N uptake of field-grown Kersting's groundnut planted in 2012. For each landrace, bars with dissimilar letters are significantly different at *p* ≤ 0.05. Error bars represent S.E.

The effect of landrace × location interaction showed that landraces Boli and Puffeun elicited greater nodule DM at Nyankpala, while Sigiri recorded much higher nodule DM at Savelugu (Figure 7A). Shoot DM of landraces Dowie, Sigiri and Boli were significantly higher at Nyankpala, while Puffeun and Funsi showed similar biomass at the two locations (Figure 7B). The shoot N content closely mirrored the shoot DM at the two locations (Figures 7B,C). However, except for landrace Sigiri, which recorded greater soil N uptake at Nyankpala, the others elicited a similar pattern of soil N uptake at the two locations (Figure 7D).

### Functional correlations between plant growth and symbiotic N nutrition

**Figure 8:**
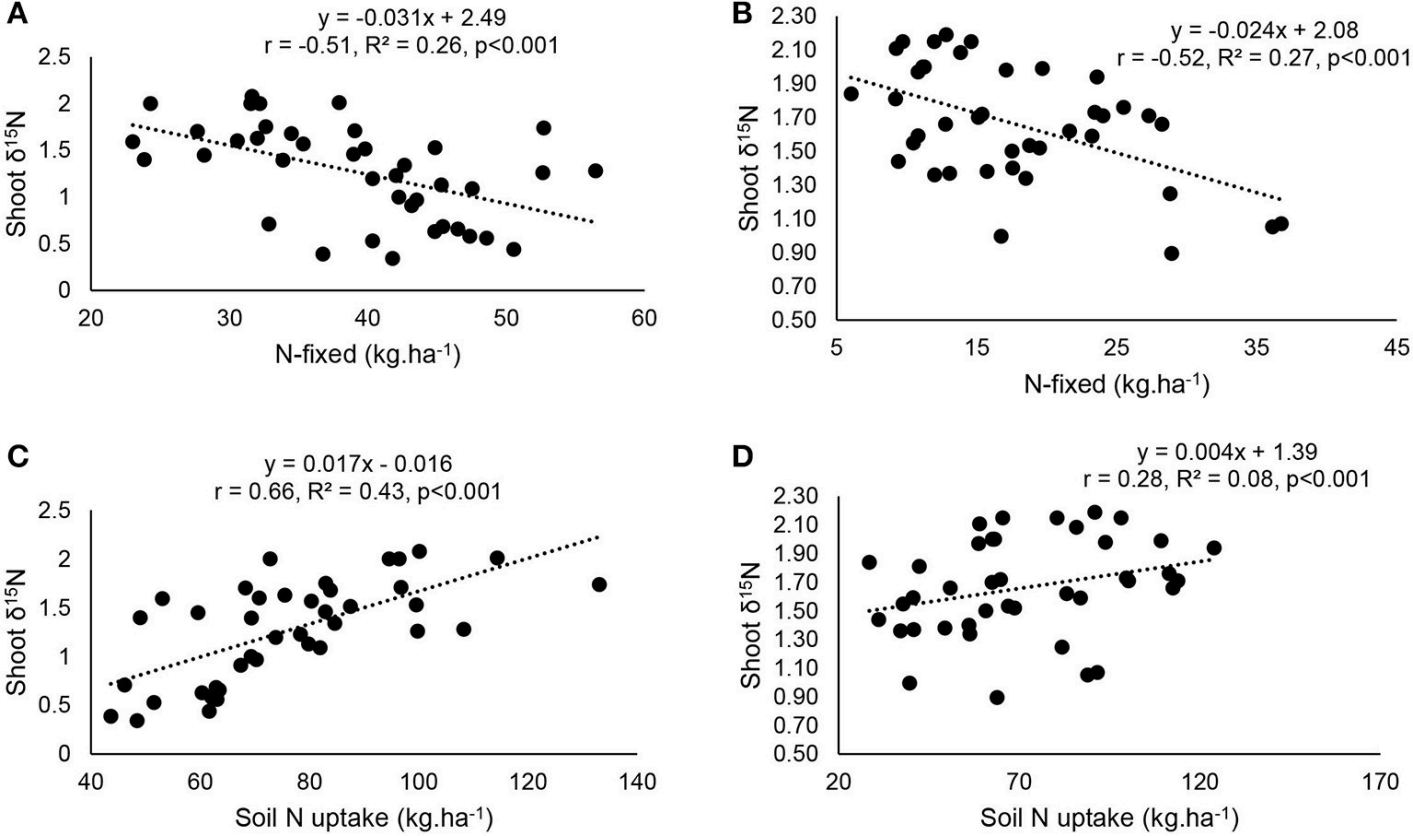
Correlation and regression analysis of **(A)** N-fixed and δ^15^N at Nyankpala, **(B)**, N-fixed and δ^15^N at Savelugu **(C)** soil N uptake and δ^15^N Nyankpala and **(D)** soil N uptake and δ^15^N at Savelugu, in in field-grown Kersting's groundnut landraces.

**Figure 9:**
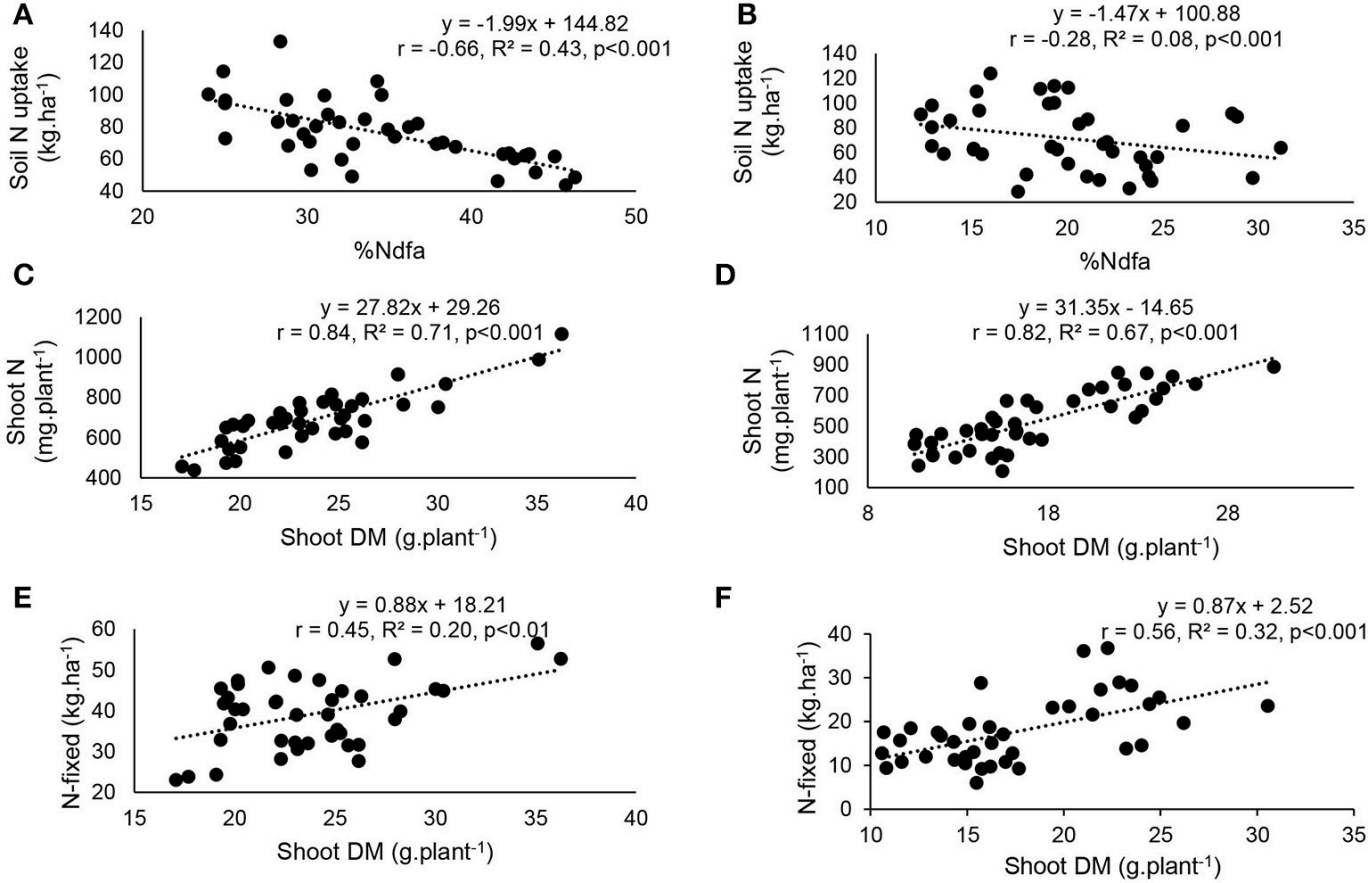
Correlation and regression analysis of **(A)** %Ndfa and soil N uptake at Nyankpala, **(B)** %Ndfa and soil N uptake at Savelugu, **(C)** shoot dry matter and N content at Nyankpala, **(D)** shoot dry matter and N content at Savelugu, **(E)** shoot dry matter and N-fixed at Nyankpala and **(F)** shoot dry matter and N-fixed at Savelugu, in field-grown Kersting's groundnut landraces.

At the two study sites, correlation and regression analyses revealed a significantly direct relationship between shoot δ^15^N and amounts of N-fixed (Figures 8A,B). The relationship between δ^15^N and soil mineral N uptake (Figures 8C,D), as well as soil N uptake and percent N derived from fixation (Figures 9A,B) was also significant for both Nyankpala and Savelugu. Shoot biomass also correlated positively with N content and N-fixed at the two locations (Figures 9C–F).

## Discussion

Kersting's groundnut is a neglected, under-utilized, and under-researched orphan legume known for its medicinal value and potential use as a food/nutritional security crop in Africa. The edible grain contains about 21.5% protein with 6.2% lysine and 1.4% methionine, two essential sulfur-containing dietary amino acids (Anon, 1979). Water extract prepared from the grain flour is often used for traditional treatment of diarrhea. Despite these useful biological traits, Kersting's groundnut remains an orphan crop with an endangered status. Although a few studies have been conducted on Kersting's groundnut (Amuti, 1980; Dakora et al., 1992; Dakora, 1998; Aremu et al., 2006; Ajayi and Oyetayo, 2009; Dansi et al., 2012; Ayenan and Ezin, 2016), field data on its nodulation and N_2_ fixation are still lacking. Little information therefore exists on its symbiotic functioning with both native and introduced rhizobia, an aspect much needed for the conservation, management and utilization of this endangered species in the African savanna.

The SSR sequence analysis showed that the Kersting's groundnut landraces studied were genetically different. The successful cross-genus transferability of SSRs in this study revealed the existence of evolutionary closeness between cowpea and Kersting's groundnut. The effective nodulation of Kersting's groundnut plants by the cowpea-nodulating strain CB756 originally isolated from *Macrotyloma africanum* (Blumenthal and Staples, 1993) showed the possibility of cowpea-Kersting's groundnut synteny. Synteny between cowpea and other legumes such as soybean (*Glycine max* L. Merr.) and clover (*Medicago truncatula* L.) were earlier reported (Lucas et al., 2011). The observed evidence of cross-taxa SSR amplification in this study could helpful in designing repeat-based primers for further use in Kersting's groundnut breeding programs.

The findings of this study also revealed effective symbiosis between the five Kersting's groundnut landraces and the native rhizobia present in Ghanaian soils. These observations are consistent with an earlier report which showed that effective nodulation of Kersting's groundnut by native rhizobia led to substantial production of grain yield (Bayorbor et al., 2010). In this study, the better nodulation and symbiotic performance at Nyankpala relative to Savelugu was evidenced by the lower δ^15^N values, higher %Ndfa, shoot N accumulation and the amounts of N-fixed at Nyankpala, which together led to increased plant growth at that site (Table S2). Applying *Bradyrhizobium* strain CB756 as inoculant to field-grown Kersting's groundnut produced variable response with regards to shoot growth, nodulation, and symbiotic functioning. Even the B values obtained from inoculation of landraces Puffeun and Funsi with the commercial *Bradyrhizobium* strain CB756 differed for whole plants, though similar at the organ level. These findings are consistent with earlier reports which showed variable response of legume genotypes to rhizobial inoculation under both field and glasshouse conditions (Gyogluu et al., 2017; Kyei-Boahen et al., 2017; Mbah and Dakora, 2017). In this study, an increase in nodulation, whether by native soil rhizobia or the introduced strain CB756, did not always translate into better plant growth, higher N_2_ fixation, and/or greater grain yield. Although increased nodule mass is generally expected to promote plant growth presumably from effective symbiosis, legume response to rhizobial inoculation in the field can be complex, and is influenced by inter-strain competitiveness between the introduced strain and native rhizobia for nodule occupancy, as well as by the differences in N_2_-fixing efficacy between native rhizobia and inoculant strain (Catroux et al., 2001; Batista et al., 2015). The findings of this study could suggest that the nodule occupants were less effective in N_2_ fixation, hence the poor symbiotic performance even with greater nodule dry matter. However, we also observed that, despite the low nodule dry matter yield with *Bradyrhizobium* inoculation at Nyankpala, shoot biomass, shoot N content, amount of N-fixed and soil N uptake were all increased (Figures 6A–E). Clearly, these unexpected results suggest the need for more field studies to increase our understanding of the phylogenetic and functional diversity of microsymbionts nodulating Kersting's bean in Africa. Such studies could also reveal the factors shaping their distribution across contrasting environments (Jaiswal et al., 2016).

Without doubt, field inoculation of Kersting's groundnut with *Bradyrhizobium* strain CB756 produced varied results. The variations in legume response to inoculation have been reported by earlier studies (Hafeez et al., 2000; Ulzen et al., 2016). The fact that only landraces Funsi and Dowie (but not the other three) accumulated greater shoot biomass at Nyankpala with inoculation suggests that they benefited from enhanced N nutrition via symbiosis, the soil, or both. The same could be said of the greater shoot biomass of inoculated landrace Funsi at Savelugu. Soil N uptake by inoculated Funsi landrace at Nyankpala (105 kg N·ha^−1^) and Savelugu (107 kg N·ha^−1^) and the amounts of N-fixed at Nyankpala and Savelugu (50 and 23 kg·ha^−1^, respectively) clearly indicate that this landrace obtained more N from soil than symbiosis for meeting its N demand. So, the significant shoot biomass of the inoculated Funsi landrace was largely due to soil N uptake than symbiotic N nutrition from bradyrhizobial inoculation. The opposite result was obtained at Savelugu, where uninoculated Kersting's groundnut landraces markedly accumulated more shoot biomass than their inoculated counterparts largely due to greater soil N uptake than symbiotic N nutrition. The five Kersting's groundnut landraces therefore exhibited low levels of symbiotic N_2_ fixation and N contribution at both Nyankpala and Savelugu due partly to soil N inhibition of N_2_ fixation. The growth of Kersting's bean plants was therefore highly complemented by soil N uptake and symbiotic N_2_ fixation, as evidenced by the significant correlations obtained between shoot N content and shoot biomass at the two experimental sites.

The occurrence of inoculation benefit (if any) was location-specific. Despite reduced nodulation by inoculation at Nyankpala, shoot DM, N content, amounts of N-fixed and soil N uptake were all greater in inoculated than uninoculated Kersting's groundnut plants at the site (Figures 6A–E). The opposite was however found at Savelugu, where the same parameters were higher in the uninoculated compared to inoculated landraces despite the lower nodulation by uninoculated plants. These findings indicate possible differences in the compatibility of Kersting's groundnut landraces with the indigenous rhizobia at the two study sites.

In addition to location effect, there was also an observed genotypic response to inoculation by the five landraces. For example, inoculated plants of Puffeun at Nyankpala recorded the lowest shoot δ^15^N, followed by uninoculated Dowie and Funsi landraces, and then inoculated plants of Boli. As a result, those treatments (in that order) showed the highest N derived from fixation. These findings suggest that, while the Puffeun and Boli landraces might have been more compatible with the introduced *Bradyrhizobium* strain CB756, the landraces Dowie and Funsi were by contrast, more compatible with native rhizobia in the Ghanaian soils studied. As a result, those strain/host combinations produced greater symbiotic N than the others.

Whether at Nyankpala or Savelugu, plant growth and various symbiotic parameters were physiologically interlinked. For example, high shoot δ^15^N values indicated lower amounts of N-fixed due to greater soil N uptake by the legume (Figure 8). Even though endogenous soil N (i.e., total N in soil) was lower at Nyankpala (0.04%) than Savelugu (0.06%), its uptake by Kersting's groundnut landraces inhibited N_2_ fixation, as evidenced by the decreasing %Ndfa with increasing soil N uptake at the two study sites (Figures 9A,B). This observation is consistent with earlier reports which showed that N_2_ fixation in legumes was suppressed by higher soil N levels (Zheng et al., 2016; Mbah and Dakora, 2017). In fact, soil N uptake and N from symbiosis complemented each other in supporting plant growth at both Nyankpala and Savelugu, as indicated by the significant correlations found between plant growth and N-fixed, N content as well as soil N uptake.

Although N_2_ fixation was increased by bradyrhizobial application to some Kersting's groundnut landraces (see Figures 5C,D for Puffeun, Sigiri and Boli), inoculation had no overall effect on grain yield of the five test landraces at the two study sites. Instances of increased N_2_ fixation in landraces were not matched by an increase in grain yield. At Nyankpala, for example, inoculated plants of Puffeun derived 45% of its N from symbiosis (as opposed to 30% by uninoculated plants) and contributed 43 kg N·ha^−1^ (compared to 28 kg N·ha^−1^ by uninoculated plants; see Figure 5), yet there were no differences in grain yield. This could imply that landraces that fixed less N were overcompensated with greater soil N uptake, which nullified the effect low N_2_ fixation on grain yield. Although the landrace × inoculation interaction was not significant for grain yield, a close scrutiny of the data from Nyankpala showed that grain yield of Puffeun was curiously higher in uninoculated (1,582 kg ha^−1^) compared to inoculated plants (1,387 kg ha^−1^). The same could be said of the landrace Puffeun at Savelugu. However, the fact that the total N of uninoculated plants of Puffeun was 111.4 kg N·ha^−1^ as opposed to 56.6 N·ha^−1^ in inoculated plants at Savelugu could account for the greater grain yield in the uninoculated plots.

It was interesting to note that unimproved Kersting's groundnut landraces could produce grain yield ranging from 1,137 to 1,556 kg ha^−1^ at Nyankpala, and 921 to 1,192 kg ha^−1^ at Savelugu. This clearly suggests that with improvement, this grain legume could outyield cowpea and Bambara groundnut in the African continent (Belane and Dakora, 2010; Mohale et al., 2014). The observed high grain yields despite lower symbiotic dependence observed in this study could suggest that Kersting's bean is a resource-use efficient crop with potential for use as a component of sustainable, low-input cropping systems. No doubt, plant growth, N nutrition and grain yield of the five landraces was influenced by location, genotype, and genotype-soil microbiome compatibility. The better plant growth, N accumulation and grain production at Nyankpala was more likely due to adequate soil moisture from the relatively higher rainfall at that site when compared to Savelugu. However, an observed transient waterlogging at the Savelugu site with rainfall could also have affected plant growth, N_2_ fixation, and grain yield when compared to Nyankpala. Of the landraces tested, Funsi exhibited increased plant growth, lower δ^15^N, higher %Ndfa, greater amounts of N-fixed, higher shoot N accumulation and increased soil N uptake at both study sites independent of inoculation. Irrespective of the location, landraces Boli and Puffeun produced the most grain yield, and Dowie the lowest. Landraces Boli and Puffeun would therefore seem to be the best candidates for future crop improvement involving field tests with many more compatible and competitive inoculant strains in multilocation trials.

Taken together, our data show that Kersting's groundnut is a very low N_2_-fixer, with levels of N derived from fixation ranging from 28 to 45% at Nyankpala, and 15 to 29% at Savelugu. Symbiotic N contribution was also low, and ranged from 28 to 50 kg N-fixed·ha^−1^ at Nyankpala, and 12 to 32 kg N-fixed·ha^−1^ at Savelugu. The amounts of N-fixed by Kersting's groundnut in this study are much lower when compared to symbiotic N contribution by cowpea, Bambara groundnut and soybean in Africa (Belane and Dakora, 2010; Mohale et al., 2014; Mapope and Dakora, 2016). There is therefore a need to identify well-adapted local rhizobial strains that can be used as inoculants to competitively increase N_2_ fixation and grain yield in this species. Our study is the first detailed report on the symbiotic N nutrition (δ^15^N, %Ndfa and amounts of N-fixed) of field-grown Kersting's groundnut in response to inoculation with *Bradyrhizobium*. Clearly, the variable inoculation response obtained in this study suggests the need for more studies to increase our understanding of the phylogenetic and functional diversity of microsymbionts nodulating Kersting's bean and other native legumes in Africa.

## Author contributions

MM: conducted field experiments, analyzed and interpreted data, and prepared the manuscript; SJ: analyzed molecular data and helped to write the manuscript; ES and BA: helped in field experiments; FDD: funded and coordinated the research and helped in manuscript preparation.

### Conflict of interest statement

The authors declare that the research was conducted in the absence of any commercial or financial relationships that could be construed as a potential conflict of interest.
