# Supplementary material for: Symbiotic N2 Fixation and Grain Yield of Endangered Kersting's Groundnut Landraces in Response to Soil and Plant Associated Bradyrhizobium Inoculation to Promote Ecological Resource-Use Efficiency
Source: Front Microbiol. 2018 Sep 11;9:2105. doi: 10.3389/fmicb.2018.02105 (PMC6142881; doi:10.3389/fmicb.2018.02105)
Supplement: Supplementary file 1 [file Table_1.docx]

# **TABLE S1** SSR primer pairs, band sizes and annealing temperatures

| **SSR primer** | **Sequence** | **Band size (bp)** | **Annealing temperature (^o^C)** | **Reference** |
| --- | --- | --- | --- | --- |
| CLM0014 | F: CGTTCACCCATTTCTCATTC  R: CAAGATCACATCCAAGCACA | 300 | 56.1 | (Xu et al., 2010) |
| CLM0018 | F: GCACATGCTCCTCAAGATTT  R: TCCTCGAAGAACTTGTCCAG | NA | 56.1 | “ |
| CLM0022 | F: GTCCACAAATCAGATGCACA  R: AGTTCCCTTCCCTTCATGTT | 210 | 50.1 | “ |
| CLM0028 | F: TTGCCTTGTTAGGTGAGAGC  R: GTGACGCGGAAAAACTCTAA | NA | 50.1 | “ |
| CLM0030 | F: AGAATTGTCCCTCCCAAGAC  R: TCTGATAACCCCAAAAGCTG | 200 | 50.1 | “ |
| CLM0033 | F: TCCAACAGGTTCTGGTCATT  R:AGAGATGATCGGAGGGATTC | 140 | 50.7 | “ |
| CLM0034 | F: ACAACCACAACCAACACCTC  R:TGAGAGAAGAAGCGGAATTG | 170 | 50.7 | “ |
| CLM0037 | F: TGCACTTCATTAGGGTCCAT  R: AGGCTATCCCTTCTCGTGTT | NS | 50.7 | “ |
| CLM0040 | F: TCAACAACAGCAACATGGAC  R: AGATGTATCGCGAAAACTGC | 200 | 50.7 | “ |
| CLM0044 | F: TGCCTTAACTCGGTTGATTC  R: CGTACAGAGGTTCCCTGTTG | 210 | 50.7 | “ |
| VuUGM80 | F:GTGTTGATTACAAGTGTCAACGTG  R: CTTAATCCCCCACCTCTGCT | NA | 56 | (Gupta and Gopalakrishna, 2010) |
| VuUGM87 | F: GAATGGGGTGGAGAGGTCTT  R: ACTCCTCCTTCCCCTTCTTG | 300 | 56.1 | “ |

NA= No amplification; NS= non specific band

**TABLE** **S2** A three-way ANOVA of plant growth, nodulation, symbiotic parameters and grain yield of five Kersting’s groundnut landraces planted at Nyankpala and Savelugu in Ghana, with or without *Bradyrhizobium* inoculation. Values (mean ± SE) with dissimilar letters in a column are significantly different at p≤0.05 (*), p≤0.01 (**), p≤0.001 (***) or ns (not significant).

| *Location* | Shoot DM  (g plant^-1^) | Nodule DM  (mg plant^-1^) | N conc'n  (%) | N content  (mg plant^-1^) | C/N  (g g^-1^) | δ^15^N  (‰) | Ndfa  (%) | N fixed  (kg ha^-1^) | Soil N uptake  (kg ha^-1^) | Grain yield  (kg ha^-1^) |
| --- | --- | --- | --- | --- | --- | --- | --- | --- | --- | --- |
| Nyankpala | 23.83±0.58a | 7.04±0.47a | 2.91±0.05a | 692.16±22.45a | 14.51±0.23a | 1.27±0.08b | 34.42±1.03a | 39.15±1.32a | 76.21±3.14a | 1335.09±43.65a |
| Savelugu | 17.54±0.77b | 6.49±0.56b | 3.05±0.10a | 535.28±29.48b | 11.59±0.28b | 1.66±0.05a | 20.03±0.78b | 17.71±1.19b | 71.51±4.06a | 1028.83±69.89b |
| *F-statistics* |  |  |  |  |  |  |  |  |  |  |
| *Landrace (L)* | *14.01** | *18.13**** | *0.60^ns^* | *7.30**** | *0.16^ns^* | *11.44**** | *11.12**** | *13.59**** | *5.26*** | *2.58** |
| *Inoculation (I)* | *0.30^ns^* | *6.40** | *2.18^ns^* | *1.17^ns^* | *0.05^ns^* | *2.54^ns^* | *2.61^ns^* | *0.20^ns^* | *2.15^ns^* | *0.56^ns^* |
| *Location (S)* | *104.14**** | *4.84** | *1.45^ns^* | *32.89**** | *53.27**** | *56.24**** | *403.30**** | *338.31**** | *1.60^ns^* | *15.77**** |
| *L x I* | *12.86**** | *45.57**** | *2.13^ns^* | *5.00*** | *0.31^ns^* | *22.11**** | *21.04**** | *3.20** | *8.31**** | *1.75^ns^* |
| *L x S* | *5.17*** | *46.91**** | *0.25^ns^* | *3.83*** | *0.06^ns^* | *1.49^ns^* | *1.17^ns^* | *1.25^ns^* | *4.23*** | *0.35^ns^* |
| *I x S* | *24.88**** | *92.82**** | *0.79^ns^* | *15.54**** | *0.18^ns^* | *0.12^ns^* | *0.20^ns^* | *15.87**** | *12.82**** | *1.51^ns^* |
| *L x I x S* | *0.81^ns^* | *14.36**** | *1.22^ns^* | *0.48^ns^* | *0.43^ns^* | *16.05**** | *14.99**** | *8.03**** | *0.49^ns^* | *2.05^ns^* |
